# Special Issue “Artificial Intelligence in Oral Health”

**DOI:** 10.3390/diagnostics12081866

**Published:** 2022-08-02

**Authors:** Jae-Hong Lee

**Affiliations:** Department of Periodontology, Daejeon Dental Hospital, Institute of Wonkwang Dental Research, Wonkwang University College of Dentistry, Daejeon 35233, Korea; ljaehong@gmail.com

I thank all authors, reviewers and the editorial staff who contributed to this Special Issue. In recent years, an increasing body of evidence has shown a direct or indirect correlation between oral health and chronic systemic diseases, including diabetes mellitus, atherosclerosis, rheumatoid arthritis, cancer, cardiovascular disease, and other non-communicable chronic diseases, although these findings remain controversial [1,2]. Typical oral disease parameters are evaluated and assessed by dental professionals using common clinical and radiographic tools including periodontal probe, dental mirror, dental explorer, and panoramic, periapical, and bitewing radiographic images, as well as cone beam computed tomography scans in some cases [3,4]. However, these conventional methods are inherently subjective, time-consuming, and expensive and may result in the under- or overestimation of diagnostic accuracy and performance [5,6]. Despite several attempts to overcome these limitations, they remain challenging and do not provide practical benefits over conventional diagnostic methods with regard to time, cost-effectiveness, and standardization.

Artificial intelligence (AI) refers to the ability of a machine that possesses its own form of intelligence to perform tasks that require human cognition. AI-based technology has emerged as a promising approach in the healthcare domain since the 2000s [7,8]. AI and machine learning based on the digitized big data archives and computing infrastructure are revolutionizing medical practice [9]. AI assists in clinical decision making through rapid and reliable data interpretation, the automation of administrative workflows to reduce non-patient-care-related activities, and direct patient participation in monitoring their health to improve health literacy [10]. AI has led to a paradigm shift in dental science, including in restorative dentistry, oral and maxillofacial surgery, prosthodontics, orthodontics, endodontics, and periodontics [11]. In particular, AI has significantly transformed dentistry and is viewed as a promising tool to revolutionize clinical diagnosis and management of oral disease. However, the exact role of AI in the prevention, diagnosis, and management of oral disease remains controversial.

AI-based algorithms will facilitate rapid, accurate, and reliable diagnosis of oral diseases and adoption of preventive strategies, as well as prompt intervention for improved treatment outcomes. Therefore, AI scores over traditional analytics and clinical decision making techniques through unbiased, consistent, and good-quality diagnosis and treatment in clinical and epidemiological scenarios. AI is particularly useful for standardized diagnosis and treatment of oral disease, which will benefit dental professionals in clinical practice. Several AI-based deep learning architectures have already been approved by the FDA and are being applied in clinical practice. In the dental field, the usefulness of AI has been assessed for the detection, classification, and segmentation of anatomical variables for orthodontic landmarks, dental caries, periodontal disease, and osteoporosis; however, these applications are still in very preliminary stages. This Special Issue is intended to lay the foundation of AI applications focusing on oral health, including general dentistry, periodontology, implantology, oral surgery, oral radiology, orthodontics, and prosthodontics, among others.
